# Psychological adaptation of recovered individuals with COVID-19: A phenomenological approach

**DOI:** 10.1192/j.eurpsy.2022.1311

**Published:** 2022-09-01

**Authors:** A. Hamdan-Mansour

**Affiliations:** The University of Jordan, School Of Nursing, Amman, Jordan

**Keywords:** Covid-19, Descriptive Phenomenology, Psychosocial wellbeing

## Abstract

**Introduction:**

Healthcare services are primarily focusing on medical and physical treatment of COVID-19 while psychosocial and mental health needs are not considered a priority.

**Objectives:**

The purpose of this study was to explore how recovered individuals with COVID-19 adapted to their psychological and social stressors during infection period.

**Methods:**

A descriptive phenomenological approach conducted using a purposeful sample of 13 individuals recovered from COVID-19 in Jordan. Data collected using unstructured interviews.

**Results:**

Perception of being diagnosed with COVID-19 revealed to three major themes; positive learning (acceptance, avoiding social pressure, and normalizing), tolerating ambiguity (denial and seeking information and guidance), and resilience (caring family, professionals’ support, self-grieving, optimism, positive thinking, and spirituality).

**Conclusions:**

The study indicates that there is a need to integrate psychosocial and mental health care services into healthcare plans provided to individuals with COVID-19.

**Disclosure:**

No significant relationships.

